# Prevalence of thyroid dysfunction in psychiatric inpatients: A cross sectional prevalence study

**DOI:** 10.6026/973206300223259

**Published:** 2026-06-30

**Authors:** Rajeev Ranjan Raj, Surbhi Chaudhary, Firoz Sheikh, Mimansha Patel, Vikram Karande, Yugantar N Wakde, Anukriti Kumari

**Affiliations:** 1 Department of Psychiatry, Geetanjali Medical College &Hospital, Geetanjali University, Udaipur, Rajasthan, India; 2 Department of Biochemistry, Saraswati Medical College, Unnao, Uttar Pradesh, India; 3 Department of Pathology, Raipur Institute of Medical Sciences, Raipur, Chhattisgarh, India; 4 Department of Oral Pathology &Microbiology, Triveni Dental College, Bodri, Bilaspur, India; 5 Department of Dentistry, AIIMS, Rajkot, Gujarat, India; 6 Department of Biochemistry, Shri Rawatpura Sarkar Institute of Medical Sciences and Research, Naya Raipur, Chattisgarh, India; 7 Department of Oral Medicine and Radiology, School of Dental Sciences, Sharda University, Greater Noida, India

**Keywords:** Anxiety, depression, prevalence, psychiatry, thyroid dysfunction

## Abstract

Thyroid dysfunction, despite its established connection to various psychiatric disorders, is underexplored in psychiatric inpatients, particularly in relation to mood disorders like depression and anxiety. A cross-sectional analysis was performed on 124 patients at a medical college, with first-episode psychiatric disorders. Thyroid function tests (TSH, T3, T4) revealed high rates of thyroid dysfunction, particularly in non-affective and affective psychoses, with common abnormalities including low TSH and high T4. Socio-demographic factors such as age, sex and family income influenced thyroid health. Thus, we show the prevalence of thyroid dysfunction in psychiatric patients and emphasizing the need for routine thyroid function testing in psychiatric assessments to improve the management of mental illnesses.

## Background:

Thyroid hormones are critical for developmental and mature brain function and development. Hormonal activity in the brain affects the activity of firing and neural programming [1]. If disturbed, it can cause numerous symptoms of the mind. Most notable are any cuts in cognition and depression. Studies have found a strong association between thyroid hormone metabolism and mental disorders, particularly mood disorders like depression and anxiety [2]. In terms of neurophysiological mechanisms of the thyroid hormones, the impact of these hormones on the brain has been reviewed. According to research, the alteration of thyroid hormone function may be linked with psychiatric disorders [3]. However, mood disorders appear to be sensitive to changes in thyroid metabolism. There have been a lot of studies to prove dysfunction of thyroid-axis has something to do with mood disorders. Researchers showed that thyroid hormones affect the central nervous system in various ways, while changes occurring in the metabolism of thyroid hormones are often associated with psychiatric disorders, particularly mood disorders. [4]. Thyroid disorders among physical disorders are uniquely related to rapid cycling. Thyroid dysfunction can cause neuropsychiatric symptoms such as depression and mania, as well as acute psychosis and dementia [5]. As these symptoms illustrate, thyroid hormones affect the human brain and so they affect behaviour. Although the link between thyroid disorders and depression has been well established, the relationship between thyroid dysfunction and anxiety has been less systematically studied. According to certain studies, panic disorder, simple phobia and obsessive-compulsive disorder are more common in thyroid patients than in the general population [6]. Co-existence of common biochemical abnormalities may be implicated in shared pathogenesis of psychiatric/thyroid disorders. Moreover, the clinical features of thyroid disorders are also sometimes confused with psychiatric manifestations such as depression and mania [7]. Hypothyroidism has been noted to resemble melancholic depression or dementia, with TSH levels correlated with depression scores. Hyperthyroidism has been linked to an increased risk of developing bipolar disorder [8]. Even though thyroid function and mental health are associated, not much has been done in terms of research on the prevalence of thyroid dysfunction among patients with major psychiatric disorders [9]. Researchers also analysed the same but its limitations are that it is retrospective and executed on a small sample size and data on the duration of illness and timing of thyroid were not available. To fill this gap, the current study will assess and compare the level of thyroid dysfunction among patients with different psychiatric disorders in a hospital-based inpatient setting [10]. Therefore, it is of interest to determine the incidence of thyroid dysfunction in psychiatric patients and also the effect of this co-morbidity on their outcomes through effective diagnosis and treatment strategies for better detection.

## Methodology:

The study aimed to assess thyroid dysfunction among psychiatric inpatients using purposive sampling. Patients selected for the study were drug-naïve or drug-free, with a minimum of 4 weeks since discontinuing oral psychotropic medications and 8 weeks from depot preparations and were diagnosed with a first-episode psychiatric disorder according to ICD-10 DCR criteria. The inclusion criteria for cases included individuals aged between 18 and 60 years, of either sex, who provided written informed consent and who met the psychiatric diagnosis criteria. Patients under the age of 18 or above 60 years, as well as those receiving psychotropic medications, were excluded from the study. The study used several tools for data collection, including a socio-demographic and clinical data sheet, a performa for diagnosis and treatment history and thyroid function tests (TSH, T3 and T4). All patients underwent thyroid function tests during their first admission to the inpatient care unit. The thyroid function was assessed by analyzing blood samples using Enzyme Linked Immunosorbent Assay (ELISA) to measure T3, T4 and TSH levels, with normal reference ranges of 0.8-2.0 ng/mL for T3, 4.6-12 μg/dL for T4 and 0.6-3.8 μU/mL for TSH. Thyroid dysfunction was diagnosed based on these biochemical parameters. The study took place over three months, following approval from the ethical committee. The data on socio-demographic characteristics, diagnosis, age, gender and medication status were collected from participants case records. The data was then analyzed using SPSS (Statistical Package for Social Sciences) version 16.0 for statistical analysis.

## Results:

In this study, among all the patients getting admitted, only those diagnosed with first episode psychiatric disorder fulfilling the inclusion and exclusion criteria were included. Table 1 shows the patient admission census during the study period, including the total number of new and old adult OPD cases, total ward admissions and total first episode psychiatric disorder admissions. During the study period, out of 1346 ward admissions, 396 patients were admitted with first episode psychiatric disorders. Table 2 shows the total number of patients admitted with various diagnostic groups during the study period, with major representation from non-affective psychosis, followed by affective psychosis and substance use disorder. Table 3 shows the socio-demographic and clinical characteristics of all the patients included in the study. In present study, total 124 patients were included among whom 99 (79.8) were male and 25 (20.2%) female. Among them 110 (88.7%) were Hindus by religion and 94(75.8%) belonged to rural area. 61(49.2%) were married, 65(52.4%) were employed and 80(64.5%) with family income below Rs. 10,000/- month. Table 4 shows various diagnostic groups among study population. The sample had major representation from Non-affective psychosis 75(60.5%) followed by affective psychosis 27(21.8%) and then substance use disorder 18(14.52%). Table 5 shows that there was almost equal frequency of patients with abnormal thyroid profile among different diagnostic group non-affective psychosis, affective psychosis and substance use disorder respectively. Among 75 patients of Non-affective patients 39(51.84%) patients presented with deranged thyroid profile. Similarly, out of 27 patients who were diagnosed with affective psychosis 14(52.0%) were having abnormal thyroid profile. Almost (50%) *i.e.* 9 out of 18 patients diagnosed with substance use disorder presented with deranged thyroid profile. Patients falling in other diagnostic group were very less hardly one representative each from personality disorder, epilepsy, dissociative and somatoform disorder so percentage of sample presenting with abnormal thyroid profile won't give a real picture. As shown in Table 6, thyroid function abnormalities were observed among the study participants in various forms. Low TSH values suggestive of hyperthyroid status were observed in 17 patients, including 6 patients with concomitant high T4 levels and 11 patients with isolated low TSH levels. High T4 levels were the most common abnormality, observed in 22 patients, while low T3 levels were seen in 8 patients.

## Discussion:

The thyroid dysfunction among psychiatric inpatients study discussion covers a comprehensive overview of the thyroid abnormalities within this population. It makes a connection between psychiatric conditions with thyroid hormones. The findings of this investigation offer important insights that can influence clinical practice, particularly with respect to thyroid function testing in psychiatry. In summary, we compare our current study with previous studies in this field and present overlaps and differences found. Previous studies have shown the association between thyroid dysfunction and immoral psychiatric illness. Research studies like Hage and Azar (2012) [11] back the theory that brain function is highly dependent on thyroid hormones, not to mention their significant relationship with mood disorders such as depression and anxiety. Yang *et al.* (2021) [12] reviewed thyroid hormones can alter the central nervous system which can lead to symptoms such as fatigue, poor cognitive function, or mood disorders (dysthymia, major depression, anxiety, etc.). This study confirms that the association between thyroid disorders and psychopathy, in particular affective disorder, is true according to these earlier studies. Analysis performed by Hassan *et al.* (2024) [13] shows a great relationship between these two disease groups implying that specialist treating depression or anxiety should should check thyroid function of patients. According to the current study, abnormal thyroid parameters were present in most of the psychiatric patients with non-affective psychosis, affective psychosis and substance use disorder. In particular, the present study shows the rate of abnormal thyroid function test, that is, elevated TSH or low T4 levels mixed with different diagnoses which is similar to Wu *et al.* study (2023) [14]. Kumar *et al.* (2026) [15] had also studied the relationship of thyroid dysfunction with the major psychiatric disorders and similarly saw the relationship of thyroid dysfunction with schizophrenia and bipolar disorder. The current study also contributes to this literature, noting that patients with affective psychosis and non-affective psychosis are especially prone to thyroid irregularities. This further bolsters the theory about a shared biochemical pathway between thyroid disorders and psychiatric disorders. But this study goes one step further and tries to fill a gap in research regarding thyroid dysfunction in inpatients with psychiatric illnesses. Even though thyroid dysfunction and psychiatric disorders have been previously linked, there has not been much of an evaluation of thyroid function of psychiatric inpatients [16]. According to the researchers, while retrospective studies suffer from a small sample size and the timing of taking the thyroid level, this study is prospective and has a large sample size. This provides strong support for performing routine thyroid function tests as part of psychiatric work-up, which is not something done all the time during clinical practice [17]. The findings of this study have also added the earlier factors such as age, gender, marital status, etc., by the socio-demographic factors which also contribute to thyroid dysfunction. Earlier studies have mainly built upon the relationship noted between thyroid dysfunction and a set of psychiatric disorders, ignoring these factors. On the other hand, unlike the previous study the current study included these socio-demographic variables to get a better understanding of the thyroid health of the psychiatric patients. Moreover, the analysis of the different types of thyroid dysfunction specifically low TSH with high T4, high TSH with low T3 etc., gives a breakdown of the actual size of the various thyroid abnormalities among the psychiatric inpatients as covered in this study. It's a very important aspect because the approach to treatment of the different types of thyroid disorders varies, especially when psychiatric medications may affect thyroid function. The elaborate categorization of thyroid dysfunction in the current study provides a new insight into how these abnormalities affect psychiatric population, which area has not been studied extensively in earlier literature. A limitation of the study was that it did not include patients who were on psychotropic medication and affects the thyroid. Earlier studies also highlighted that the use of psychiatric medication may affect the levels of thyroid hormones.

## Conclusion:

We show the high prevalence of thyroid dysfunction among psychiatric inpatients, particularly in non-affective psychosis, affective psychosis and substance use disorders. Routine thyroid function testing should be incorporated into psychiatric assessments to prevent misdiagnosis and improve treatment outcomes. Further research is needed to explore the impact of thyroid dysfunction on psychiatric symptoms and treatment responses.

## Figures and Tables

**Table 1:** Patient admission census during the study period

**Total no. of new adult cases in O.P.D.**	**3921**
Total no. of old adult cases in O.P.D.	11330
Total no. of admission in ward	1346
Total no. of first episode psychiatric disorder admitted	396

**Table 2:** Total no. of patients admitted with various diagnostic groups during the study period

**Non-Affective psychosis**	**448**
Affective psychosis	347
Substance use disorder	266
*Other	10
*Other (diagnostic group) included patients diagnosed with personality disorder,
epilepsy, dissociative &somatoform disorder.

**Table 3:** Socio-demographic variables of the sample (N=124)

**Variables**	**Groups**	**FREQUENCY (n)**	**PERCENT (%)**
	Male	99	79.8
Gender	Female	25	20.2
	Married	61	49.2
Marital Status	Unmarried	63	50.8
Religion	Hindu	110	88.7
	Muslim	9	7.3
	Other	5	4
Residence	Rural	94	75.8
	Urban	30	24.2
	Illiterate	33	26.6
Education	Up to High School	56	45.16
	Above High School	35	28.22
Occupation	Employed	65	52.4
	Unemployed	59	47.6
Family	<Rs.10,000/-month	80	64.5
Income	Rs.10,000-15,000/-month	31	25
	Rs.15,001 and above	13	10.48

**Table 4:** Various diagnostic group of the sample (N=124) included in the study

**Diagnosis**	**Frequency (n)**	**Percentage (%)**
Non-affective Psychosis	75	60.5
Affective Psychosis	27	21.8
Substance use disorder	18	14.52
Other	4	3.2
Total	124	100

**Table 5:** Frequency of abnormal thyroid parameters among sample (N=124)

**Diagnostic group**	**Abnormal**	**Abnormal**	**Abnormal**
	TSH	T3	T4
	n (%)	n (%)	n (%)
Non affective Psychosis (N=75)	14/75 (18.67)	4/75 (5.33)	21/75(28.00)
Affective Psychosis (N=27)	6/27 (22.22)	2/27 (7.40)	6/27 (22.22)
Substance use disorder (N=18)	5/18 (27.78)	2/18 (11.11)	2/18 (11.11)
Other (N=4)	0/4 (0)	1/4 (25.00)	2/4 (50.00)
Total (N=124)	25/124 (20.16)	9/124 (7.25)	31/124 (25.00)

**Table 6:** Frequency of thyroid dysfunction among sample (N= 124).

**Thyroid abnormality**	**n (%)**
Low TSH, High T4	6 (4.84)
Low TSH	11 (8.87)
High TSH	4 (3.22)
High T4	22 (17.74)
Low T3	8 (6.45)
Low T3, High T4	1 (0.80)
Low TSH, LowT4	1 (0.80)
